# Phylogenetic analysis and biological characteristics of an Akabane virus isolated in China

**DOI:** 10.3389/fvets.2025.1691476

**Published:** 2025-10-27

**Authors:** Gan Li, Shikai Cai, Xiyu Liu, Hairui Li, Limei Qin, Dengshuai Zhao, Junjie Huang, Ping Li, Yuanhang Zhang, Yajie Zheng, Miaomiao Zhang, Han Gao, Wenqiang Tang, Xialing Zhao, Bin Shi, Wanxiang Qi, Mengmeng Zhao, Keshan Zhang

**Affiliations:** ^1^Guangdong Provincial Key Laboratory of Animal Molecular Design and Precise Breeding, School of Animal Science and Technology, Foshan University, Foshan, China; ^2^Institute of Animal Science, Xizang Academy of Agricultural and Animal Husbandry Sciences, Lhasa, China

**Keywords:** Akabane virus, genetic variation, phylogeny, antigenic epitope, pathogenicity

## Abstract

Akabane virus (AKAV) is an arbovirus that can cause miscarriage, premature birth, congenital malformations, and encephalomyelitis in young livestock. It is currently widely prevalent in China. Vero and MDBK cells were utilized to determine the virus titer and growth curve of the AKAV FS202301 strain in this study. Subsequently, the viral solution was intracranially (IC) or intraperitoneally (IP) inoculated into 8-day-old suckling mice, and the pathogenicity was explored by observing clinicopathological changes, and hematoxylin–eosin (HE) staining. Additionally, the S, M, and L segment sequences of the FS202301 strain were analyzed, phylogenetic trees were constructed, and antigenic epitopes were predicted to investigate its genetic variation. The results revealed that the viral titer of strain FS202301 was 10^6^ TCID_50_/mL, with the number of viral copies peaking 24 h post-infection (hpi). This strain predominantly induced damage to the central nervous system, culminating in the death of suckling mice. It was classified as belonging to the genogroup Ia, exhibiting the highest degree of variation in the M segment, lower degrees of variation in the S and L segments, and no recombination events in any of the genes. The Gc protein encoded by the M segment contains more amino acid mutation sites and predicts a greater number of antigenic epitopes. This study aims to enhance the understanding of AKAV genetic variation in China and to establish a theoretical foundation for the future prevention and control of AKAV epidemics.

## 1 Introduction

Akabane disease (AKAD) is an insect-borne disease caused by the Akabane virus (AKAV) that leads to abortions, premature births, stillbirths, central nervous system damage in newborn fetuses, as well as congenital arthrogryposis-hydranencephaly syndrome in pregnant sheep and cows, and it is mainly transmitted by midges and mosquitoes (1–4). AKAV is classified within the family *Bunyaviridae* and the genus *Orthobunyavirus*, a single-stranded negative-stranded RNA virus characterized by a capsid, and a genome comprising three segments: S, M, and L (5, 6). The S segment is highly conserved and encodes the nucleoprotein (N) as well as the nonstructural protein (NSs); the M segment is highly variable and encodes a precursor polyprotein that is post-translationally cleaved to produce the viral envelope glycoproteins (Gn and Gc) and the nonstructural protein (NSm); and the L segment encodes solely the viral RNA polymerase (L protein) (7). Based on the M segment sequences, AKAV was classified into four distinct groups (I-IV), with genogroup I further subdivided into two subgroups: Ia and Ib (8). Kono et al. (8) demonstrated that AKAV genogroup Ia is more neuronophilic and is capable of causing encephalomyelitis in calves, as well as reproductive disorders in pregnant cows.

In 1959, AKAV was first isolated in Gunma Prefecture, Japan, and was subsequently reported in Australia, Israel, Indonesia, South Korea, and China (9, 10). Although no large-scale outbreak of AKAV has been documented in China to date. However, Wang et al. (11) conducted serological surveys across 24 provinces in China from 2006 to 2015, revealing that cattle and sheep tested seropositive for AKAV antibodies in most regions. This indicates a widespread prevalence of AKAV infection among cattle and sheep in China. In 1992, Liao et al. (12) isolated 11 strains of AKAV from dairy farms in Taiwan, China, where abortion and fetal malformations had been observed in pregnant cows, marking the first report. In 2010–2011, Cao et al. (13) isolated two strains of AKAV (HN10169 and HN10174) from mosquitoes, representing the first documented occurrence of AKAV isolation from field insects in China. Between 2013 and 2016, five novel AKAV strains were isolated from bamboo rats in Guangxi Province, China (14). In 2016, the AKAV strain TJ2016 was isolated from bovine serum in China, and sequence analysis of the S and M segments indicated that it belonged to genogroup II (15). In 2019, Tang et al. (10) successfully isolated the AKAV strain GXDH 01 from goat blood, with phylogenetic analysis indicating that this strain is classified within genogroup Ia. Furthermore, it has been demonstrated that AKAV can infect a variety of animals, including cattle, sheep, pigs, bamboo rats, camels, deer, and horses (10, 14, 16–19), resulting in significant economic losses to the global animal husbandry industry. This study presents an analysis of the genetic evolution and pathogenicity of the AKAV FS202301 strain (GenBank accession no. PQ567126-PQ567128) isolated and preserved in the lab (20), aimed at examining the genetic variation of this strain and providing a theoretical foundation for advancing molecular epidemiology and detection research related to AKAV.

## 2 Materials and methods

### 2.1 Virus titer determination

The virus solution of the FS202301 strain, which has been plaque-purified, was diluted to a 10-fold dilution and inoculated into a 96-well plate containing a monolayer of Vero cells, with 100 μL added to each well. For each dilution level, seven duplicate wells were set up, and a negative control was included to ensure the accuracy and reliability of the experiment. Following incubation at 37 °C in a 5% CO_2_ environment for 5 to 7 days, the number of wells exhibiting cytopathic effects (CPE) at each dilution was observed using a microscope. This experiment was conducted three times, and the half tissue culture infectious dose (TCID_50_) of the virus was calculated using the Reed-Muench method (21).

### 2.2 Determination of growth curves

The virus solution (infection complex MOI = 1) was inoculated into MDBK cells, and harvest viral RNA at 10 time points ranging from 0 to 84 h post-infection (hpi). The quantity of viral copies at each time point was quantified using the RT-qPCR assay. Viral proliferation curves were plotted using GraphPad Prism 9.1.0 (GraphPad, San Diego, CA).

### 2.3 Genome-wide analysis of strain FS202301

Fifteen reference sequences for AKAV S, M, and L were selected from the GenBank database on the NCBI website (Supplementary Tables 1–3). Nucleotide and amino acid similarity analyses, as well as amino acid sequence alignments, were conducted on the fragment sequences of the FS202301 strain using GraphPad Prism (Version 9.0.0) and the MegaAlign function of DNAStar software (Supplementary Figure 2).

### 2.4 Phylogenetic analysis

Phylogenetic analyses were performed on 225 selected AKAV S sequences, 108 AKAV M sequences, and 44 AKAV L sequences (Supplementary Tables 4–6). Phylogenetic trees were constructed using the maximum likelihood (ML) method in MEGA software (version 11.0.13, Mega Limited, Auckland, New Zealand). The ML method was repeated 1,000 times with bootstrap, while the remaining parameters were maintained at default values. The resulting phylogenetic trees were then imported into the online beautification editing software (The Interactive Tree of Life)^1^ for subsequent sorting and editing.

### 2.5 Recombination detection

Preliminary identification of potential recombination events in the collected AKAV S, M, and L fragments was conducted using seven algorithms available in RDP software (version 4.0): RDP, GENECONV, BootScan, MaxChi, Chimera, SiScan, and 3Seq. The results of the analysis indicate that strains exhibiting four or more positive (+) results with a significance level of *p* < 0.05 are classified as recombinant strains.

### 2.6 Prediction of N, Gn, NSm, and Gc protein antigenic epitopes

Antigenic epitopes of N, Gn, NSm and Gc proteins of AKAV FS202301 strain were predicted using an online prediction site for antigenic epitopes.^2^

### 2.7 Pathogenicity study in suckling mice

To evaluate the pathogenicity of the FS202301 strain, 8-day-old suckling mice were divided into three groups (n = 8 per group), two of which were inoculated intracranially (IC) and inoculated intraperitoneally (IP) with 10 or 100 μL of 10^6^ TCID_50_/mL viral fluids, and the negative control (NC) was injected with phosphate buffer solution (PBS). The mental status of suckling mice was monitored daily following inoculation. Suckling mice in the IC group were dissected at 1, 3, and 5 day post inoculation (dpi), while those in the IP group were dissected at 2, 5, 8, and 11 dpi to assess the pathological conditions of various organs (Euthanasia was performed on suckling mice by cervical dislocation). Following dissection, tissue specimens, including brain, heart, liver, spleen, lung, kidney, and intestine, were collected for pathological analysis. All experimental procedures were conducted as described by Gao et al. (22). Total RNA was extracted from brain, heart, liver, spleen, lung, kidney, and intestine tissues of suckling mice using TRIzol reagent (Sangon Biotech, Songjiang, Shanghai, China). RNA samples were reverse transcribed using Hiscript® III All-in-One RT SuperMix Perfect for qPCR (Vazyme, Nanjing, China). Primer sequences were as follows: forward (F): 5’-TAAGACGCCACAAC CAAGTGT-3′, reverse (R): 5’-CCGAAATGCGATGGAGCGTA-3′. The PCR reaction conditions included pre-denaturation at 95 °C for 2 min, followed by 40 cycles of amplification (95 °C for 10 s, 60 °C for 30 s). The AKAV *NSS* gene was selected as the target for viral genome copy number detection. This experiment received approval from the Animal Ethics Committee of Foshan University.

## 3 Result

### 3.1 Virus titer and determination of growth curve

After 48 h of the virus solution was inoculated with Vero cells, the virus titer of FS202301 strain was measured to be 10^6^ TCID_50_/mL. By evaluating viral replication levels across distinct time intervals, we demonstrated that viral replication levels of the FS202301 strain exhibited an exponential increase from 0 to 24 hpi in MDBK cells, reaching a peak at 24 h. Subsequently, the viral copy number gradually declined and stabilized after 72 h (Supplementary Figure 1).

### 3.2 Genome-wide analysis of strain FS202301

Nucleotide and amino acid similarity analyses were conducted on each fragment of the FS202301 strain using GraphPad Prism (Version 9.0.0) and the MegaAlign function of the DNAStar software (Supplementary Figure 2). The results indicated that the S segment of strain FS202301 exhibited the highest nucleotide similarity (99.6%) with strain HN10169-2010 and the lowest nucleotide similarity (81.9%) with strain AN_9398–2018; the highest amino acid homology (99.6%) was observed with strains DHL10M110–2010, GD18240-China-2018 and HN10169-2010, while the lowest amino acid similarity (88.9%) was with AN_9398–2018 strain. The M segment of strain FS202301 exhibited the highest nucleotide and amino acid similarity with the HN10174-2010 strain, at 99 and 98.9%, respectively. Conversely, it displayed the lowest nucleotide and amino acid similarity to strain MP496-1972, at 70.2 and 74.3%. In contrast, the L segment of strain FS202301 demonstrated the highest nucleotide and amino acid similarity to strain HN10174-2010 (99.4 and 99.8%), the lowest nucleotide similarity to strain CS0016-1975 (86.2%), and the lowest amino acid similarity to strain R7949-1968 (95.9%).

The segments of strain FS202301 were analyzed and compared utilizing the MegAlign function of DNAStar software (Table 1). The results indicated that the S segment exhibited a high level of conservation, with only a single mutation identified at amino acid 221 (T211A). In contrast, the M segment harbored 40 amino acid mutations, with the Gc protein exhibiting the highest number of mutation sites, followed by the NSm protein, while the Gn protein exhibited the fewest mutation sites. Additionally, seven amino acid mutations were identified in the RdRp protein encoded by the L segment.

**Table 1:** Mutation sites for S, M and L segments.

Gene	Mutation sites
S	T211A
M	V104I, D163E, T277I, T300A, T323I, Q364R, H386D, I391T, I409V, N496S, F513L, I526T, V584A, M586V, S598G, V602I, R614G, F618L, S704P, D709S, N710S, M736T, G761S, V780I, M778A, D799N, T838A, A866D, E875V, V929I, G936S, T966A, M1080L, A1201T, H1230Y, A1288V, E1347Q, A1363T, V1387I
L	V354I, I855V, R1439S, L1752F, R1971K, K1996R, S2125T

### 3.3 Phylogenetic tree and recombination analysis

**Figure 1:**
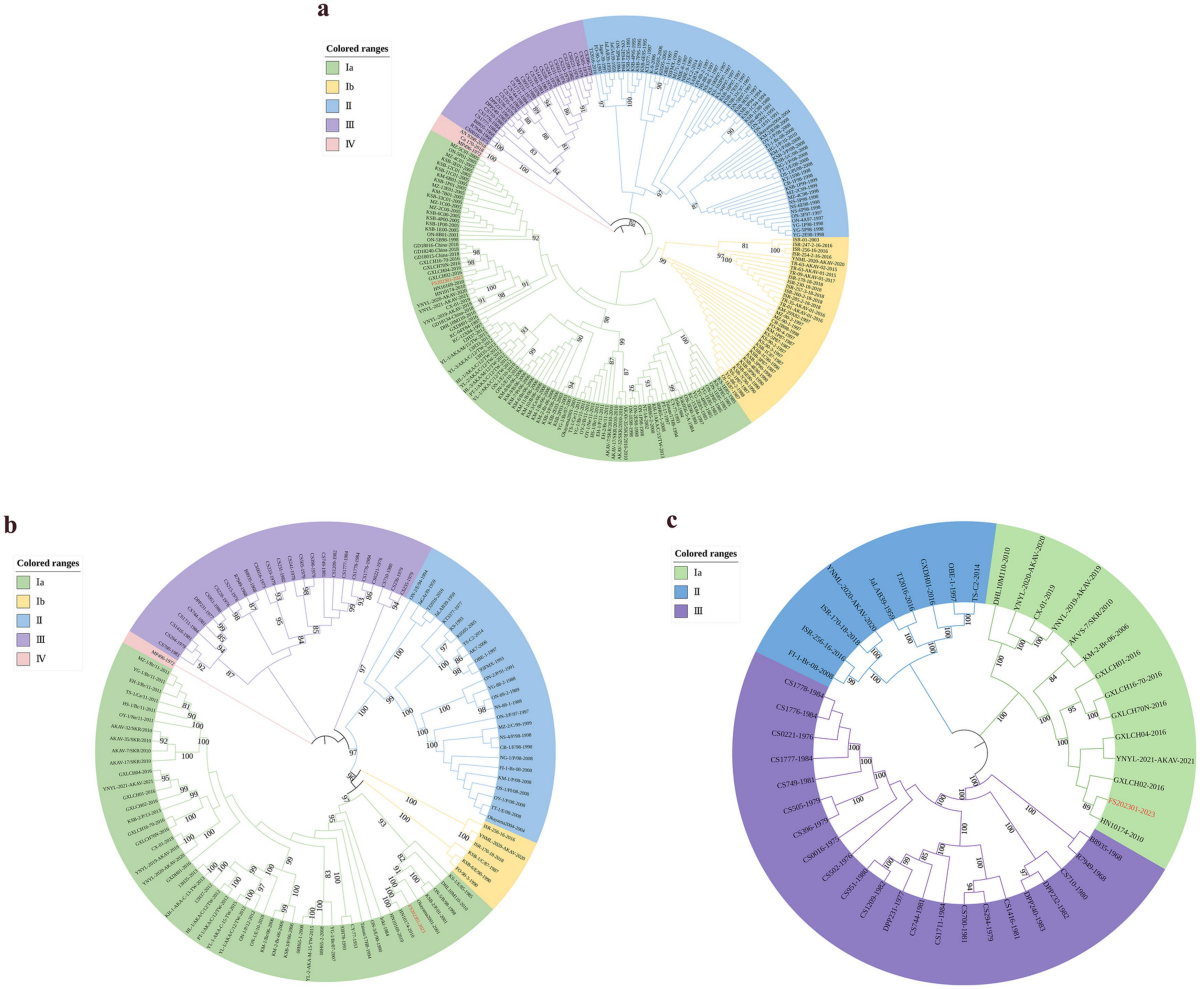
The construction of phylogenetic trees were performed using the ML method in MEGA software with 1000 bootstrap replicates. **(a)** Construct a phylogenetic tree based on the S segment. **(b)** Construct a phylogenetic tree based on the M segment. **(c)** Construct a phylogenetic tree based on the L segment.

To investigate the genetic variation of the various segments of the FS202301 strain, AKAV S, M, and L segments (225 S segments, 108 M segments, and 44 L segments) from different countries and years were selected from NCBI for genetic evolutionary analysis alongside the FS202301 strain, resulting in the construction of phylogenetic trees (Figure 1). The result revealed that the FS202301 strain is classified within genogroup Ia. In the phylogenetic trees based on the S and L segments, the FS202301 strain exhibited the closest genetic relationship to the GXLCH02-2016 strain. Conversely, in the phylogenetic tree constructed from the M segments, the FS202301 strain was found to have the closest genetic distance to the HN10714-2010 strain.

To evaluate the presence of recombination events within the segmented sequences of the isolated FS202301 strain, RDP software (version 4.0) was utilized to analyze the multiple alignment of 226 AKAV S segments, 109 AKAV M segments, and 45 AKAV L segment sequences. The results of this analysis indicated that no recombination events were detected.

### 3.4 Prediction of N, Gn, NSm, and Gc protein antigenic epitopes

Antigen epitope prediction was conducted for the N, Gn, NSm, and Gc proteins of FS202301 strain. The results indicated that 9 antigenic epitopes were predicted on the N protein, 5 on the Gn protein, and 7 on the NSm protein, while approximately 21 antigenic epitopes were identified on the Gc protein (Supplementary Figure 3). The Gc protein exhibited the highest potential for antigenic epitopes.

### 3.5 Pathogenicity study in suckling mice

**Figure 2:**
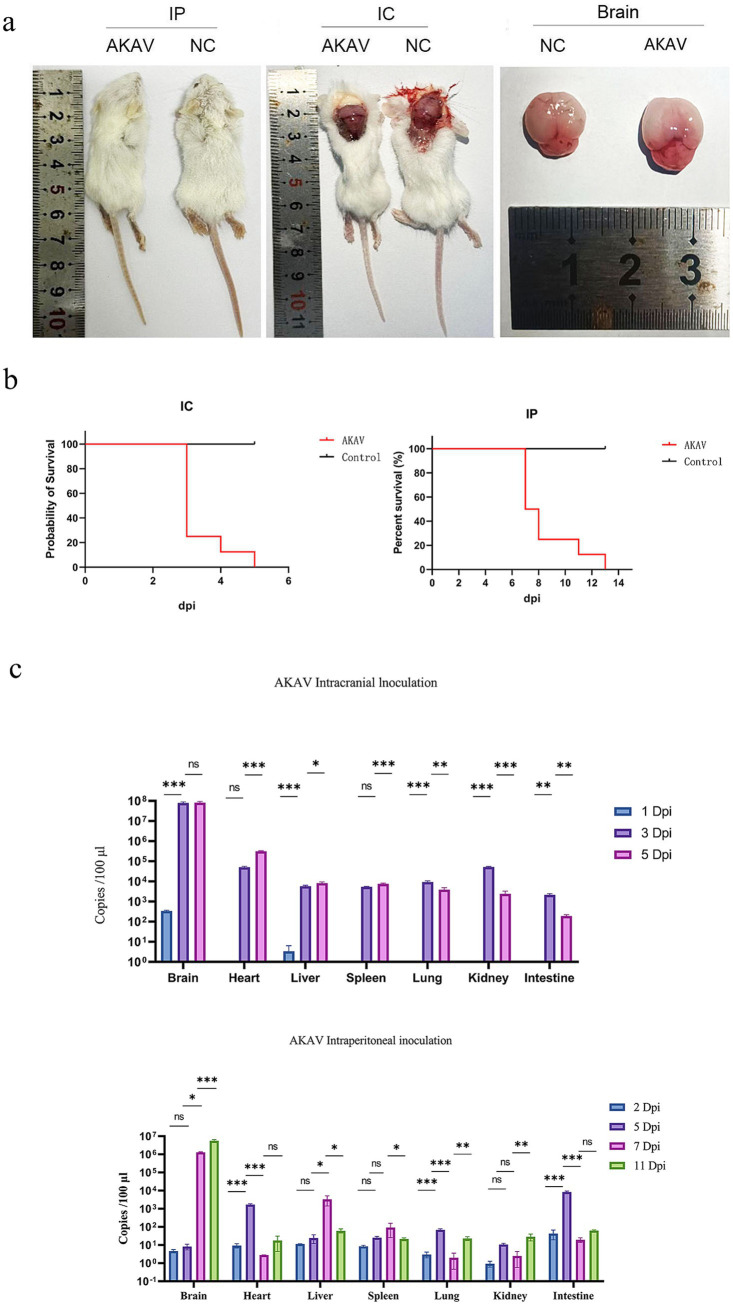
Pathogenicity assessment of FS202301 isolate in suckling mice: mortality, viral load dynamics, and tissue tropism. **(a)** Postmortem examination of suckling mice; **(b)** Survival curves of suckling mice; **(c)** Viral load dynamics in various organs of suckling mice at different time points. The data are shown as means ± SD (error bars). Asterisks (*) indicate a significant difference between groups (ns, *p* < 0.12; *, *p* < 0.033; **, *p* < 0.002; ***, *p* < 0.001).

Following IC inoculation, six suckling mice exhibited symptoms such as anorexia, lethargy, ataxia, and moribund state by 3 dpi, with the remaining mice were in a state of moribund by 5 dpi. After 6-day post-IP inoculation, the suckling mice gradually exhibited symptoms such as depression, ataxia, incontinence, and anorexia. By 7–8 dpi, six suckling mice were near death, one was in a state of moribund by 11 dpi, and by 13 dpi, the remaining suckling mice were all in a moribund state (Figure 2b). Moribund suckling mice were euthanized and dissected. Gross pathological examination revealed that both IC and IP groups exhibited primary lesions localized to the brain, characterized by cerebral edema, pallor of brain tissue, effacement of cortical sulci, and smoothing of cerebral gyri (Figure 2a).

RT-qPCR targeting the *NSs* gene was conducted on samples collected from the brain, heart, liver, spleen, lungs, kidneys, and intestines of suckling mice (Figure 2c). In the IC group, AKAV was detected exclusively in the brain and liver at 1 dpi, while viral presence was observed in all examined tissues (brain, heart, liver, spleen, lungs, kidneys, and intestines) at 3 and 5 dpi. Quantitative analysis revealed that the viral load in the brain was significantly higher than in other tissues, followed by the heart. In contrast, the IP group showed nearly undetectable viral levels at 2 dpi, followed by elevated viral loads in the intestines and heart at 5 dpi. By 8 and 11 dpi, the brain exhibited significantly higher viral loads compared to other tissues, with the heart showing the second highest levels, while remaining tissues maintained low viral loads. Brain tissue was selected as the representative organ for AKAV detection. The quantitative analysis demonstrated distinct viral replication patterns between the two groups: in the IC group, brain viral loads peaked at 3 dpi before gradually declining, whereas in the IP group, brain viral loads showed a rapid increase starting at 5 dpi, with the rate of increase slowing by 8 dpi (Figure 2c).

**Figure 3:**
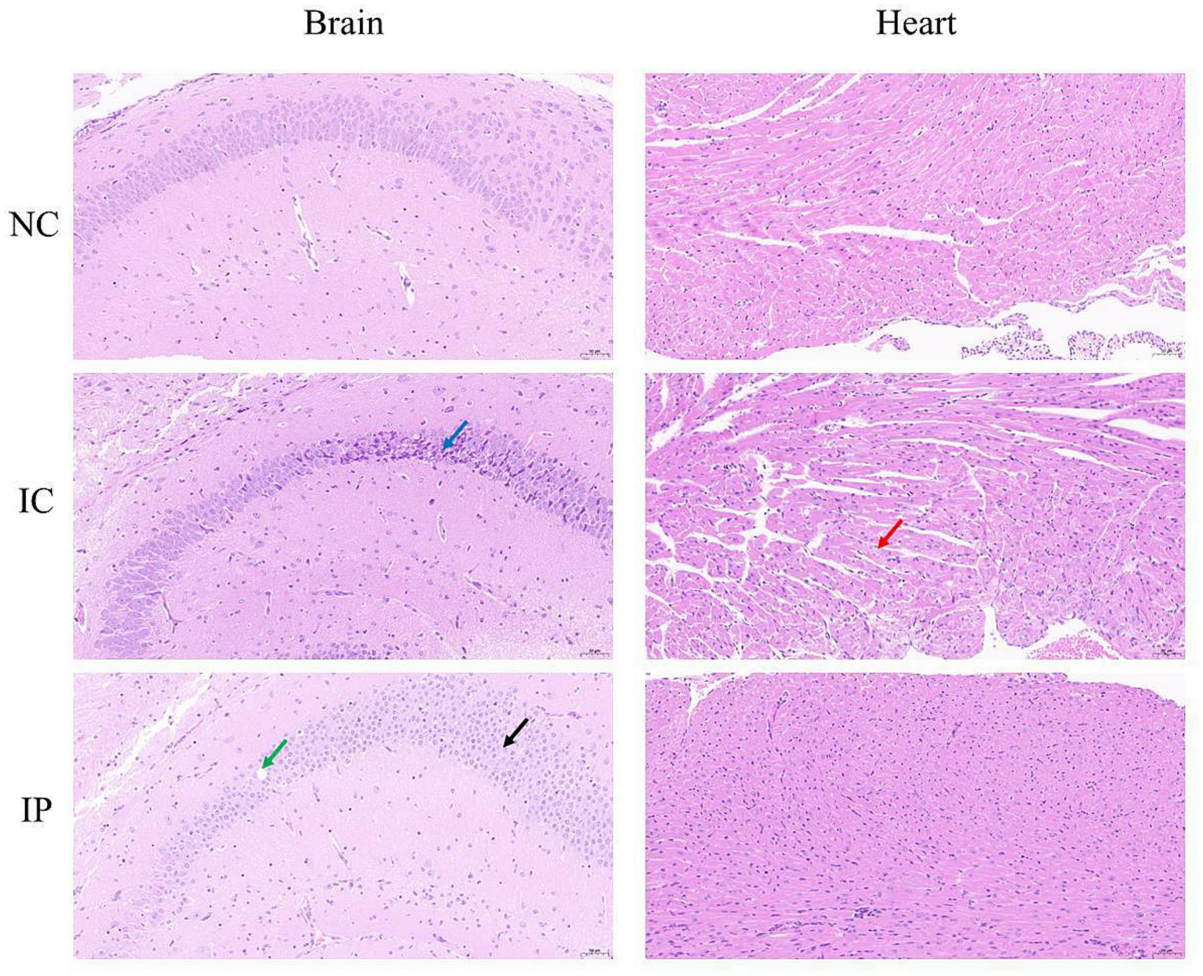
Histopathological analysis of FS202301 strain inoculated in suckling mice.

Histopathological examination through HE staining revealed distinct histopathological damage in the brains of suckling mice, which varied depending on the inoculation route (Figure 3). In the NC group, neither the brain nor the heart displayed notable pathological abnormalities. In the IC group, histopathological examination revealed distinct alterations in brain tissue architecture. Within the hippocampal formation, neurons in the CA2 region and dentate gyrus (DG) exhibited morphological changes, characterized by cellular shrinkage (indicated by dark blue arrows), increased basophilia, and loss of distinct nuclear-cytoplasmic boundaries. Similarly, a subset of neurons in the hypothalamus and brainstem demonstrated comparable pathological features. Cardiac tissue showed mild hydropic degeneration of cardiomyocytes (marked by red arrows), swollen cells, sparse and lightly stained cytoplasm and interstitium, including connective tissue and blood vessels, without obvious necrosis or other abnormalities. In the IP group, histopathological analysis revealed distinct neuropathological alterations. Focal neuronal necrosis (indicated by black arrows) was occasionally observed in the hippocampal CA1 region, thalamus, hypothalamus, and brainstem, characterized by nuclear fragmentation and pyknosis. The hippocampal CA2 region exhibited sporadic vacuolar degeneration in pyramidal cells (marked by green arrows), manifesting as rounded cytoplasmic vacuoles. Notably, no significant pathological changes, such as cardiomyocyte degeneration or necrosis, were observed in the myocardial tissue.

## 4 Discussion

AKAV is an arbovirus primarily transmitted through Culicoides and mosquitoes (23–25), widely prevalent in tropical and temperate climate regions such as Africa, Asia, the Middle East, and Australia (22), posing a significant threat to livestock farming, particularly in cattle and sheep populations. A serological survey on Chinese cattle and sheep revealed that AKAV is widely present in China, with a significantly higher incidence observed in cattle compared to sheep and goats (22). Currently, genogroup Ia strains are predominantly identified in Japan and China, while genogroup Ib strains are primarily located in Japan and Israel; genogroup II strains are mainly distributed in Japan, genogroup III strains are chiefly observed in Australia, and genogroup IV strains are primarily found in South Korea (15, 26). Notably, genogroup Ia strains exhibit the highest prevalence, resulting in significant economic losses to the farming industry.

In this study, nucleotide and amino acid similarity analyses of the S, M, and L segment sequences of the FS202301 strain compared to the reference strain demonstrated that the nucleotide similarity of the S segment ranged from 81.9 to 99.6%, while the amino acid similarity ranged from 88.9 to 99.6%. The nucleotide similarity of the M segment ranged from 70.2 to 99%, and the amino acid similarity ranged from 74.3 to 98.9%. For the L segment, the nucleotide similarity was between 86.2 and 99.4%, whereas the amino acid similarity was between 95.9 and 99.8%. These results indicate that the nucleotide and amino acid differences in the M segment are the most pronounced, reflecting a relatively high degree of variability. In contrast, the nucleotide and amino acid similarities between the S and L segments show smaller differences, suggesting a reduced level of variation. The M segment is recognized as the genomic segment exhibiting the greatest variability among Bunyaviruses, whereas the S segment demonstrates a lower degree of variation. The RdRp encoded by the L segment, which is essential for viral genome replication and transcription, has a conserved structure (27–30). This indicates that the results of this study are consistent with existing research findings.

Phylogenetic analysis of the S, M, and L segments revealed that the FS202301 strain is classified within the genogroup Ia. Among them, phylogenetic analysis of the S segment indicated that FS202301 is genetically closest to the GXLCH02 strain, isolated in 2016. Conversely, phylogenetic analyses based on the M and L segments demonstrated that FS202301 shares greater genetic proximity with the HN10174 strain, isolated in 2010. Previous studies have demonstrated that AKAV genogroup Ia is capable of causing encephalomyelitis in calves, exhibiting a neurotoxicity that is greater than that of genogroup II strains (31). Despite the high seroprevalence of AKAV detected in China, infected animals often present with mild symptoms or remain cryptically infected, with only transient fever and mild neurological disorders observed during the peak breeding season of Culicoides and mosquitoes (22). The FS202301 strain has been classified as belonging to genogroup Ia. It was hypothesized that, similar to other strains within genogroup Ia, it may induce encephalomyelitis in infected animals. The present study examined the pathogenicity of FS202301 strain in 8-day-old suckling mice, and all of the mice exhibited severe neurological symptoms following infection, consistent with previously reported findings. Comparative analysis revealed that IC inoculation induced more rapid disease progression and greater severity compared to IP inoculation, likely attributable to the direct viral penetration of the blood–brain barrier, resulting in immediate neural tissue invasion and subsequent neurological damage. Quantitative viral load assessment across multiple organs at various time points demonstrated that the brain consistently maintained the highest viral titers, which showed progressive increase throughout the infection course. In addition, due to the different modes of virus inoculation, AKAV was detected in all organs of suckling mice in the IP group at different times, whereas in the IC group, AKAV was detected only in the brain at 2 dpi, and the rest of the organs at 3 and 5 dpi, which indicated that the AKAV was systemically infected; and the viral loads of AKAV in the IC group were higher than those of the IP group in all organs. The results of HE staining reveal that both the IC and IP groups exhibited pathological damage in the brain, further confirming the brain as a primary target of AKAV infection. These findings provide compelling evidence that the brain serves as the primary target for AKAV infection and replication.

In this study, a comparative analysis of the amino acid sequences of the segments of the FS202301 strain revealed that the M segment contained the most amino acid mutation sites, whereas the S segment exhibited the fewest. In *orthobunyaviruses*, the glycoproteins Gc and Gn, encoded by the M segment, assemble into spikes on the viral particles and play crucial roles in viral attachment, cell fusion, and the induction of host immune responses (32). The Gc protein exhibits high immunogenicity and serves as the principal target for neutralization, capable of inducing the production of neutralizing antibodies and facilitating attachment to mammalian cells; the Gn protein is involved in viral attachment to insects and other arthropods, whereas the NSm protein may be implicated in viral assembly and morphogenesis (33–35). The majority of the mutated amino acid sites in the M segment of the FS202301 strain are situated within the Gc proteins, and modifications at these sites may have significant implications for their immunogenicity. Ishihara et al. (36) constructed multiple NSm deletion mutants using reverse genetics and assessed their pathogenicity, identifying several regions that are critical for viral infectivity (amino acids 323–460, 369–447, 323–367, and 447–460). They found that the NSm protein influences AKAV replication by functioning as a virulence factor. In this study, the majority of amino acid mutations identified in the FS202301 NSm protein were located within the region encompassing amino acids 323–460 (T323I, Q364R, H386D, I391T, I409V, V460I, and N496S), hypothesized that mutations at these sites may significantly influence its pathogenicity.

The AKAV N protein is the most abundant protein in the virion and infected cells, which induces the production of complement and triggers complement fixation antibodies after infection of the animal host, and has become widely utilized in virus or antibody detection (37–39). Additionally, there are at least five antigenic regions on the Gc protein, which is the main neutralizing protein of the virus (40). In this study, the antigenic epitopes of the FS202301 strain were predicted, and it was discovered that the Gc protein exhibited the highest number of predicted antigenic epitopes, followed by the N protein. These findings provide a theoretical foundation for the development of monoclonal antibodies targeting the AKAV Gc and N proteins, as well as for future innovations in vaccine and detection methods.

In this study, the viral titer and growth curve of the FS202301 strain were assessed. The results indicated that the viral titer was measured at 10^6^ TCID_50_/mL, with the viral copy number peaking at 24 h post-infection, followed by a gradual decline and stabilization after 72 h. Pathogenicity tests conducted on 8-day-old suckling mice revealed that the FS202301 strain primarily induced neurological damage. Furthermore, phylogenetic analyses of the S, M, and L segments of strain FS202301 indicated that it is classified within genogroup Ia and exhibits a close genetic relationship with strains GXLCH02 and HN10174. The strain did not undergo recombination. Through comparative analysis of amino acid sequences and the prediction of antigenic epitopes, we identified a greater number of amino acid mutation sites and antigenic epitopes on the Gc protein. These findings provide a theoretical basis for comprehending the potential genetic variations of AKAV and for the development of monoclonal antibodies in China in the future.

## Data Availability

The datasets presented in this study can be found in online repositories. The names of the repository/repositories and accession number(s) can be found in the article/Supplementary material.

## Footnotes

^1^ https://itol.embl.de

^2^ http://tools.immuneepitope.org/bcell/
