# Supplementary material for: Phylogenetic analysis and biological characteristics of an Akabane virus isolated in China
Source: Front Vet Sci. 2025 Oct 27;12:1691476. doi: 10.3389/fvets.2025.1691476 (PMC12597804; doi:10.3389/fvets.2025.1691476)
Supplement: Supplementary file 7 [file Image_1.pdf]

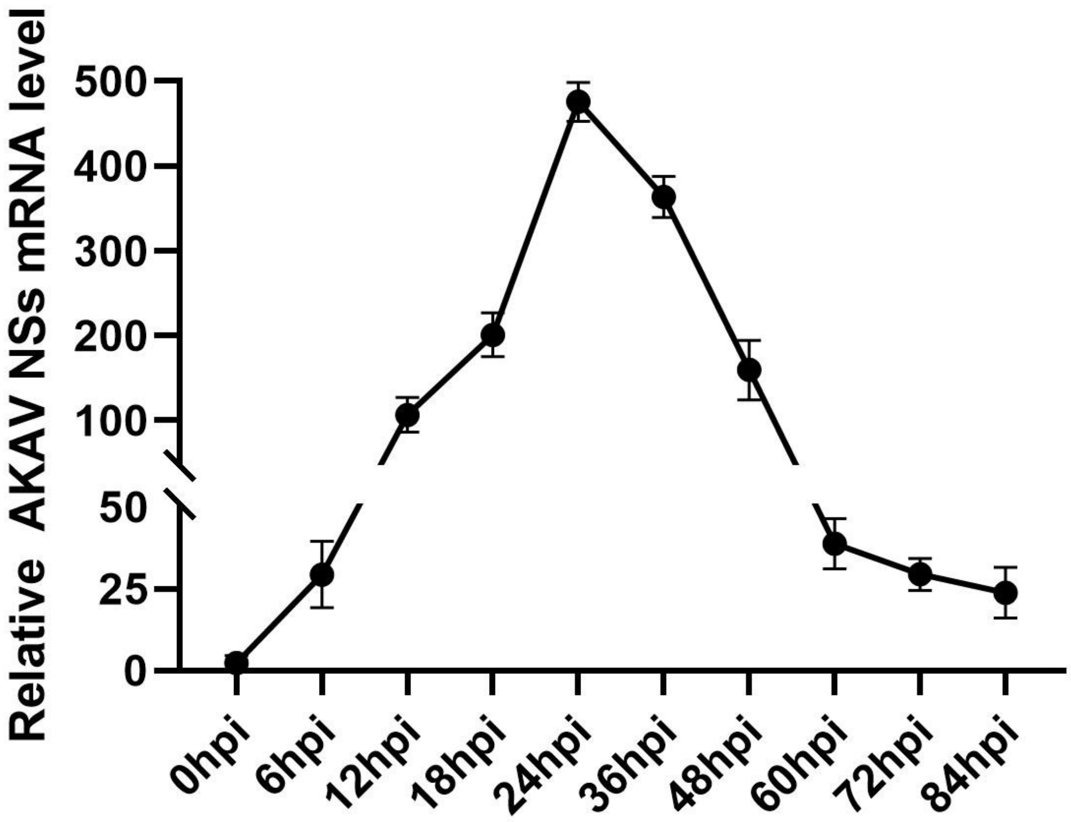

Supplementary Figure 1 The replication levels of the *NSs* gene mRNA of AKAV at different time periods.

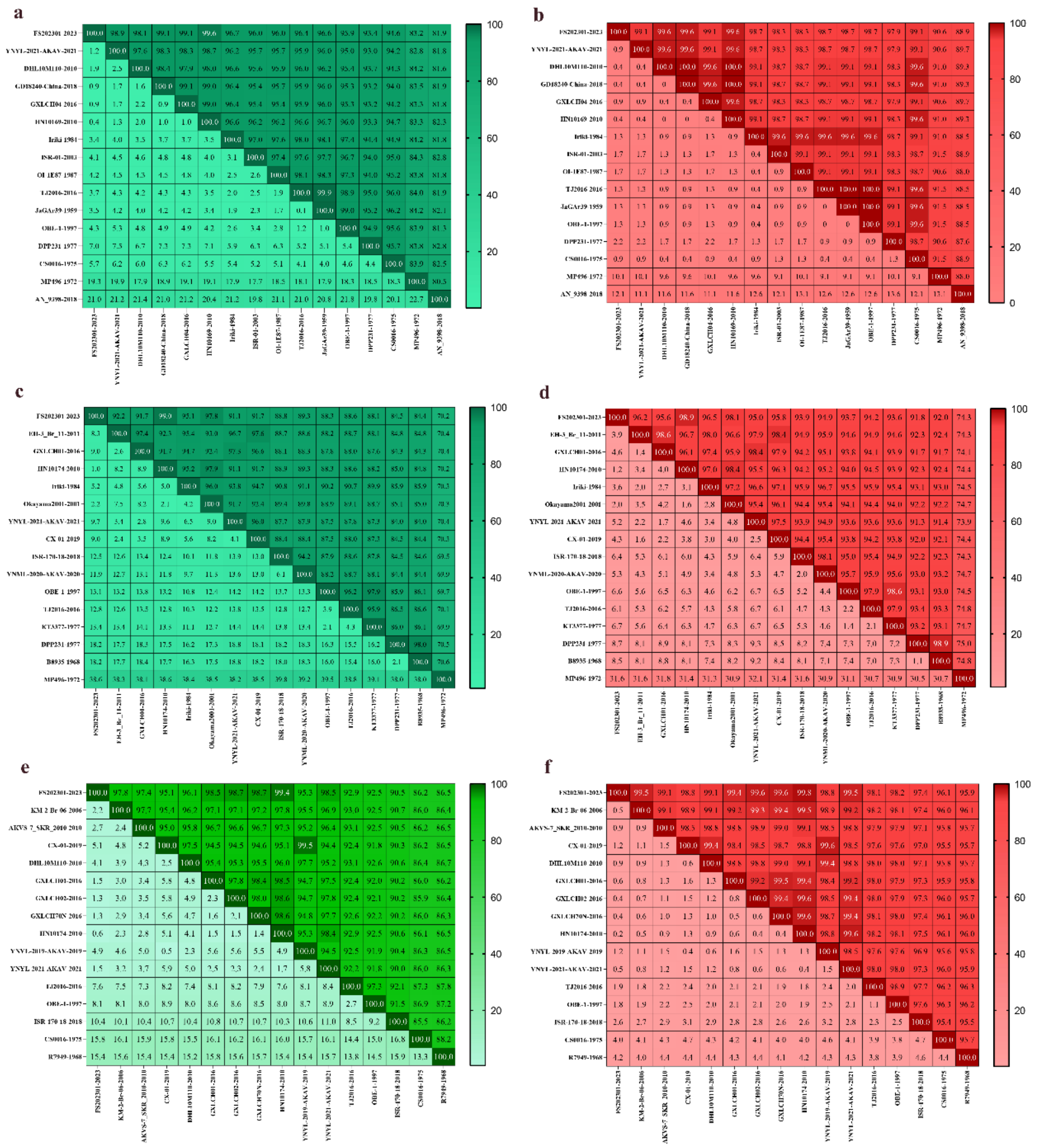

Supplementary Figure 2 Analysis of nucleotide and amino acid similarity of different fragments of the FS202301 strain. a. Nucleotide similarity analysis of the S segments; b. Amino acid similarity analysis of the S segment; c. Nucleotide similarity analysis of the M segment; d. Amino acid similarity analysis of the M segment; e. Nucleotide similarity analysis of the L segment; f. Amino acid similarity analysis of the L segment.

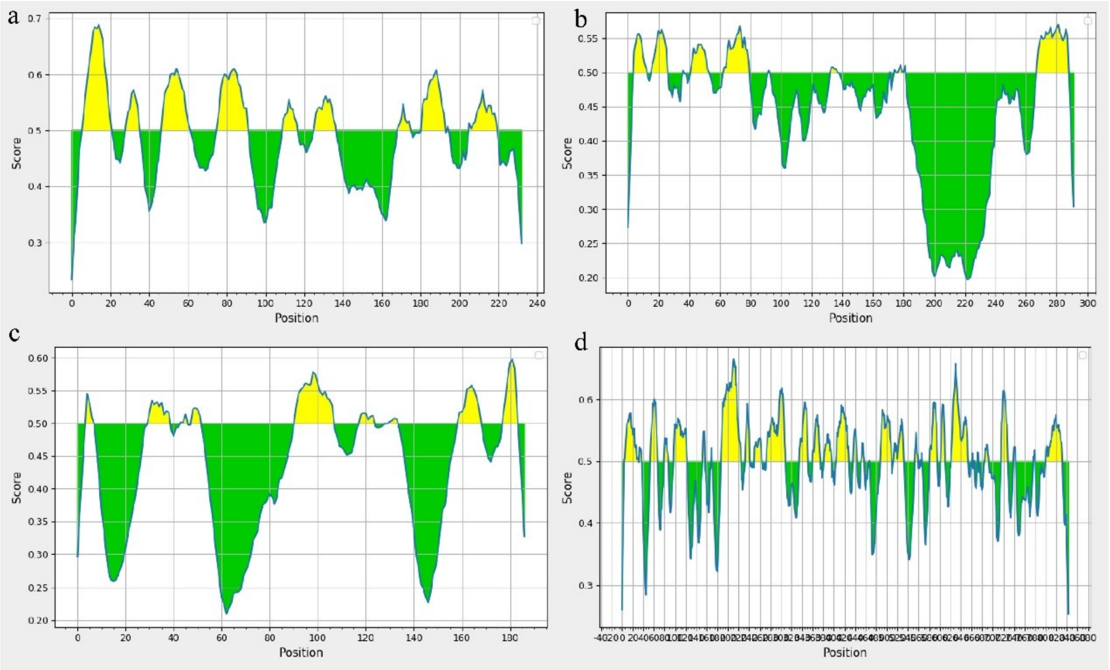

Supplementary Figure 3 Prediction on the antigenic epitopes of N, NSm, Gn, and Gc proteins in strain FS202301. a. N protein antigen epitope prediction; b. Gn protein antigen epitope prediction; c. NSm protein antigen epitope prediction; d. Gc protein antigen epitope prediction.
